# A case of dysphagia associated with an increase in antipsychotic dosage

**DOI:** 10.1192/j.eurpsy.2025.2106

**Published:** 2025-08-26

**Authors:** M. Velasco Santos, M. A. Morillas Romerosa, P. Vázquez Giraldo, I. Diéguez De Juan, C. Serrano Fuentes, F. Mir Biribay, H. Rincón Reques, G. García Cepero

**Affiliations:** 1 Psychiatry, Hospital Universitario La Paz (Madrid), Madrid, Spain

## Abstract

**Introduction:**

Dysphagia is a rare but significant side effect of antipsychotic medications, often associated with extrapyramidal symptoms. These adverse effects become particularly relevant when dosages are increased, as seen with various antipsychotics. This report describes a case involving a 57-year-old male with a long-standing diagnosis of schizophrenia who developed dysphagia following an increase in amisulpride dosage. The patient was admitted to the psychiatric unit due to psychotic decompensation after interrupting his treatment regimen of aripiprazole 30 mg/day and amisulpride 200 mg/day for two weeks. His psychotic relapse was also precipitated by the hospitalization of his critically ill mother. Following his admission, his previous treatment was reintroduced, and the amisulpride dose was increased to 600 mg/day, with diazepam 15 mg/day added. Subsequently, he developed bradykinesia, tremor, rigidity, and oral dysphagia, necessitating a liquid diet. Despite the introduction of biperiden 4 mg/day, symptoms persisted. Comprehensive physical and neurological examinations, blood tests, and a cranial computed tomography (CT) scan were performed, revealing no abnormalities other than the parkinsonian-like symptoms. An otolaryngological assessment indicated that the dysphagia was likely of neurological origin and probably related to the use of antipsychotic medications.

**Objectives:**

To present a case of dysphagia and parkinsonian symptoms triggered by an increase in amisulpride dosage in a patient with schizophrenia, highlighting the importance of careful monitoring during dose adjustments.

**Methods:**

This is a case report describing a single patient. The methodology involves a detailed examination of this unique case, including clinical evaluation, diagnostic testing, treatment modifications, and outcomes.

**Results:**

Due to the persistence of symptoms and their temporal association with the increase in amisulpride dosage, amisulpride was discontinued and switched to olanzapine 10 mg/day. Gradual improvement in both parkinsonian symptoms and dysphagia was noted over the subsequent days. Dysphagia resolved within one week, and by the time of follow-up, the patient had resumed a normal diet and experienced significant improvement in motor symptoms.

**Conclusions:**

This case highlights the potential for amisulpride to induce extrapyramidal symptoms, including dysphagia, particularly when doses are increased. The temporal relationship between the amisulpride dose increase and the onset of symptoms, along with the resolution of symptoms after discontinuation, supports this association. Clinicians should be vigilant in monitoring for such side effects, especially in patients requiring dose adjustments of antipsychotic medications.

**Disclosure of Interest:**

None Declared

